# Increased Amygdala Response to Shame in Remitted Major Depressive Disorder

**DOI:** 10.1371/journal.pone.0086900

**Published:** 2014-01-30

**Authors:** Erdem Pulcu, Karen Lythe, Rebecca Elliott, Sophie Green, Jorge Moll, John F. W. Deakin, Roland Zahn

**Affiliations:** 1 The University of Manchester and Manchester Academic Health Sciences Centre, School of Medicine, Neuroscience and Psychiatry Unit, Manchester, United Kingdom; 2 The University of Manchester and Manchester Academic Health Sciences Centre, School of Psychological Sciences, Neuroscience and Aphasia Research Unit, Manchester, United Kingdom; 3 Cognitive and Behavioral Neuroscience Unit, D’Or Institute for Research and Education (IDOR), Rio de Janeiro, Brazil; 4 Institute of Psychiatry at King’s College London, Department of Psychological Medicine, Centre for Affective Disorders, London, United Kingdom; University of Melbourne, Australia

## Abstract

Proneness to self-blaming moral emotions such as shame and guilt is increased in major depressive disorder (MDD), and may play an important role in vulnerability even after symptoms have subsided. Social psychologists have argued that shame-proneness is relevant for depression vulnerability and is distinct from guilt. Shame depends on the imagined critical perception of others, whereas guilt results from one’s own judgement. The neuroanatomy of shame in MDD is unknown. Using fMRI, we compared 21 participants with MDD remitted from symptoms with no current co-morbid axis-I disorders, and 18 control participants with no personal or family history of MDD. The MDD group exhibited higher activation of the right amygdala and posterior insula for shame relative to guilt (SPM8). This neural difference was observed despite equal levels of rated negative emotional valence and frequencies of induced shame and guilt experience across groups. These same results were found in the medication-free MDD subgroup (N = 15). Increased amygdala and posterior insula activations, known to be related to sensory perception of emotional stimuli, distinguish shame from guilt responses in remitted MDD. People with MDD thus exhibit changes in the neural response to shame after symptoms have subsided. This supports the hypothesis that shame and guilt play at least partly distinct roles in vulnerability to MDD. Shame-induction may be a more sensitive probe of residual amygdala hypersensitivity in MDD compared with facial emotion-evoked responses previously found to normalize on remission.

*Whoever blushes confesses guilt, true innocence never feels shame.*

*JJ Rousseau*

## Introduction

The importance of excessive self-blame to the distinction between depressive emotions and healthy sadness has been recognized since Freud’s seminal observations [1]. Excessive proneness to self-blaming emotions, such as guilt [2], [3] and shame [4], occurs in episodes of major depressive disorder (MDD) and in remission. This suggests that proneness to self-blame may be a trait mechanism of continuing vulnerability to depression. Whilst standard clinical assessments do not examine shame and do not distinguish guilt from shame [5], social psychologists identify distinct cognitive components [6]. Shame entails the imagination of how others perceive oneself [7], [8], whereas guilt depends on internal moral evaluation [8]. It is a matter of debate, however, whether people can distinguish between guilt and shame and which components are captured by currently employed self-report measures [6] [3], [9], [10]. Differential neural responses to different basic emotion categories such as sadness and fear have been observed in MDD, consistent with different pathophysiological roles for these emotions [11]. Using the same approach to moral emotions, showing distinct neural activation patterns associated with shame and guilt in MDD would provide key evidence for their different pathophysiological roles. This would have important clinical implications by highlighting the need to refine future clinical assessments of self-blaming emotions in order to improve the accuracy of diagnostic criteria.

A deeper understanding of the role of different self-blaming feelings in the psychopathology of MDD requires the consideration of their distinctive qualities and social functions as outlined in a previous paper [3]. Shame has been shown to involve feeling that one has been lowered in the esteem of others [12], is related to external comparison and competition [13] and its characterological nature is thought to make it particularly maladaptive. In contrast, guilt has been linked with failing to live up to internalized moral duties [12]. Proneness to self-blaming emotions has mostly been assessed using questionnaire measures aimed at the underlying emotions as hidden constructs by asking for the hypothesized behavioural consequence of the emotion (e.g. hiding/withdrawal for shame and reparative action for guilt) rather than probing participants’ subjective intuitions about these emotions which clinical descriptions rely on. This was based on the assumption that people are not able to distinguish emotions such as shame or guilt well [6]. Recent work on the neural basis of moral emotions [14], however, has shown that participants exhibit distinctive neural signatures to be associated with stimuli subjectively reported as evocative of a particular moral emotion [15], [16]. This is in keeping with anthropological evidence of transcultural ubiquity of distinct moral emotions [17] that must rely on transculturally stable conceptual underpinnings [18]. The subjective experience of guilt in healthy control groups has most consistently been associated with fMRI activation of medial frontopolar [19]–[24] and septal-subgenual cingulate areas [19], [21], [22], [25]. The evidence on neural signatures of shame in healthy populations is scarce. The only fMRI study using stringent statistical methods, was unable to identify shame-selective brain activations when comparing with guilt [26]. Other fMRI studies have investigated embarrassment [20], [23], an emotion primarily directed at preserving one’s own social reputation rather than blaming oneself for failure as entailed in shame [27]. Evidence from social psychological research suggests that both embarrassment and shame involve imagining an observer, whereas guilt does not [27]. Mental imagery is known to activate brain areas involved in sensory perception [28], [29]. Embarrassment was indeed associated with heightened activation in areas linked to sensory perception, such as the visual cortex, when compared with guilt in one study [23]. Interestingly, imagined intentional violations of social norms elicited amygdala responses in one study, but it was not measured whether people felt guilt or shame [30].

To our knowledge, the only neuroimaging study of self-blaming emotions in MDD found normal Blood-Oxygenation-Level-Dependent (BOLD) effects in fronto-limbic regions in people remitted from symptoms [31]. This study modeled trials that were most strongly associated with guilt. Shame-associated trials, however, were not modeled and it is thus unknown whether the fMRI responses to shame were distinctly altered in MDD.

Here, we used fMRI in order to investigate whether there are distinctive neural signatures of shame relative to guilt in people with MDD remitted from symptoms. By choosing a carefully matched control group with no personal or family history of MDD, resulting group differences can be interpreted as associated with trait vulnerability factors for MDD [32]. Furthermore, by studying people with remitted MDD, we were able to equate the levels of distress and emotional intensity linked to shame- and guilt-related stimuli presented during fMRI between groups.

We hypothesized (i) that the neural response to shame could be distinguished from that to guilt within the fronto-temporo-limbic networks previously associated with moral emotions [33], [34], and (ii) that people with MDD would show heightened neural responses to shame compared with the control group. More specifically, we expected shame to activate regions linked to sensory perception of emotions more strongly than guilt. This is based on the prediction that shame entails mental imagery of critical observers, whereas guilt is experienced in the absence of imagined external observers and thus less dependent on external perceptual systems [27]. Further, there is solid evidence in the non-social visual imagery literature, that mental imagery activates areas representing sensory experiences [28], [29]. It is thus reasonable to assume that social mental imagery involves brain regions linked to perception of external stimuli of social relevance. The amygdala is one of the key regions involved in the perception of emotionally and socially relevant stimuli, such as facial expressions [35]–[38]. The amygdala has not been found to be activated for guilt [19], [22], [25], [26]. Whilst there are several interpretations for this absence of guilt-related activation, one possible explanation is that guilt does not involve a great degree of external sensory perceptual simulation because it does not require simulating external observers [27] compared with shame. We therefore hypothesized that shame would be associated with stronger amygdala responses relative to guilt.

Based on the importance of a visuo-spatial mental model when simulating an observer as entailed in shame [27], but not guilt, we expected the right temporo-parietal junction to show distinctive activation for shame relative to guilt. This was based on its activation in social cognition tasks that require visuo-spatial perspective taking [39]. We further expected the posterior superior temporal sulcus to be more activated for shame relative to guilt, given the solid evidence of its involvement in the perception of socially relevant cues such as biological motion [36], [40] which could play an important part in mental models of critical observers.

## Materials and Methods

### Participants

#### Ethics statement

This study was approved by the South Manchester NHS Research Ethics Committee. Informed consent was obtained from all participants (oral for phone pre-screening and written for subsequent stages). Oral consent for phone pre-screening was documented on an anonymised phone pre-screening questionnaire. Oral consent for phone pre-screening was approved by the Ethics Committee.

#### Inclusion/exclusion of participants

Participants were part of a larger clinical research project and recruited using online and print advertisements. Initial suitability was assessed with a phone pre-screening interview (described in [31]). Participants in the MDD group fulfilled criteria for a past major depressive episode according to Diagnostic and Statistical Manual IV-TR [41], and for a moderate to severe depressive episode according to the International Classification of Diseases-10 with at least 2 months duration requiring treatment and remission of symptoms for at least 12 months. Exclusion criteria were current axis-I disorders and a history of alcohol or substance abuse or past co-morbid axis-I disorders being the likely primary cause of the depressive syndrome (see Table 1 for the clinical details of the MDD group). The healthy control group had no current or past axis-I disorders and no first degree family history of MDD, bipolar disorder, or schizophrenia.

**Table 1:** Clinical characteristics of remitted MDD group (N = 21).

***Past MDD subtype***	
With melancholic features	11/21
With melancholic & psychotic features	1/21
No specific subtype	9/21
***Number of previous MDEs***	
1	13/21
2	5/21
3	3/21
***Last MDE details***	
Average length of MDE (months)	17.4±20.2 (range: 3–96)
Average time in remission (months)	20.4±17 (range:12–84)
***Antidepressant medication at time of study***	
SSRI/SNRI antidepressant	6/21
None	15/21
***Previous medication in subgroup with no medication***	
SSRI/SNRI antidepressant	10/15
SNRI and tricyclic combination	1/15
No antidepressant medication	4/15
***Life-time axis-I co-morbidity*** *	
Anorexia nervosa	2/21
Anorexia nervosa, binge-eating subtype	1/21
Anorexia nervosa and bulimia nervosa	1/21
No life-time co-morbidity	17/21
***Family history***	
First degree relative with MDD (diagnosed)	15/21
First degree relative with MDD (questionable)	2/21
Distant relative MDD	1/21
No family member with history of MDD	3/21

* All co-morbid disorders were fully remitted at time of study. None of the co-morbid disorders was a likely primary cause of the depressive episodes. SSRI = selective serotonin reuptake inhibitor, SNRI = serotonin norepinephrine reuptake inhibitor. MDD subtype classification was based on adapting the SCID-I for DSMIV-TR to allow lifetime assessment of the subtypes. All medication-free participants had stopped medication well before the required washout phase.

In total, 171 people participated in the phone pre-screening interview, N = 79 passed this screening with 36 in the remitted MDD and 43 in the control group and were invited for visit 1. Of these, 33 individuals pre-screened as remitted MDD and 30 pre-screened as control participants were reachable, able and willing to be seen on the first study day after reading the participant information sheet sent to them. After the first day of the study, 5/33 individuals from the remitted MDD group were excluded (N = 1 fulfilled criteria for current MDD, N = 2 showed residual symptoms of post-traumatic stress disorder, N = 1 had a relapse and developed a major depressive episode between the first study day and the MRI scanning date. The remaining N = 28 participants confirmed as remitted MDD underwent MRI. MRI data from 21/28 scanned participants from the MDD group could be included in the analysis (N = 2 were excluded because of head movement greater than 4 mm, 1 because of selecting more than one moral emotion in more than 5% of trials, 4 were excluded because they had less than 6% guilt or shame responses in one of the fMRI runs). All 30 participants seen on the first study day who had fulfilled phone pre-screening criteria for the healthy control group were confirmed as fulfilling inclusion and exclusion criteria on clinical assessments and were invited for MRI scanning, however, 1 was not scanned because not being reachable following the first study session, leaving 29 that were scanned. Data from 18/29 scanned control participants could be included in the final analysis (data from N = 1 was excluded because of selection of more than one feeling on more than 5% of trials, N = 1 due to abnormalities of small vessels on the MRI scan, N = 1 due to head movement greater than 4 mm, N = 2 because of signal dropouts in important ROIs: frontopolar, ventral frontal cortex and ATL, N = 6 because of less than 6% guilt or shame trials in one of the fMRI runs).

In total, 18 healthy control participants and 21 individuals with remitted MDD (15 with no current antidepressant medication) were included in the final analysis. Functional connectivity analyses related to guilt [31] and behavioral data [3] have been previously reported. fMRI data related to shame were modeled and reported in this paper for the first time. All participants had normal or corrected-to-normal vision. The groups were matched on age, gender and years of education (see Table 2 for basic demographic information). Participants were invited for a clinical interview in which psychiatric, medical and family history were assessed along with a neurological exam which was carried out by a board-certified psychiatrist (RZ). Furthermore, a Structured Clinical Interview for DSM-IV-TR (SCID-I) Mood Disorders Module A and the International Neuropsychiatric Interview which was adapted to allow assessment of lifetime axis-I disorders including substance and alcohol abuse, a shortened version of the Weissman Family History Screen, the Montgomery Asberg Depression Rating Scale (MADRS) and the Global Assessment of Functioning (GAF) scale (Axis V, DSM-IV) were employed. Both groups had MADRS scores that were well below the cut-off for depression (<10), but the remitted MDD group showed slightly higher scores. Both groups had GAF scores indicating minimal or absent symptoms (>80), although the control participants had significantly higher scores (Table 2).

**Table 2:** Group comparison on demographic and basic clinical variables.

	Control	RemittedMDD	Teststatistic	p-value
Age	22.8±3.0	25.7±7.8	t = −1. 60	.12
Education (years)	15.6±1.7	16.1±1.9	t = −. 85	.40
Gender	15 Female	17 Female	CC = .04	.85
MADRS	.2±.7	1.1±1.8	U = 144	.09
GAF	89.4±4.3	83.7±7.2	U = 97.5	.005*

CC = contingency coefficient, * = significant at p = .05 threshold, 2-tailed, control: N = 18, remitted MDD: N = 21, U = Mann-Whitney-U. A similar table has been reported in [31].

### fMRI Paradigm

Participants were presented with written statements describing actions counter to social and moral values described by social concepts (e.g. ‘stingy’, ‘boastful’) in which the agent was either the participant (“self-agency” condition, N = 90) or their best friend (“other-agency” condition, N = 90, norms for the stimuli are further described in [19], [42] and a full list of stimuli is available on request). Self- and other-agency conditions used the same social concepts (self-agency: e.g. “[participant's name] does act stingily towards [best friend’s name]”, other-agency: e.g. “[best friend’s name] does act stingily towards [participant’s name]”). 50% of trials used negative social concepts (e.g. ‘does act stingily’) and 50% used negated positive social concepts (e.g. ‘does not act generously’). In addition we used a low-level resting-state baseline condition: fixation of visual pattern with no button press (N = 90). Stimuli were presented in an event-related design for a maximum of 5 seconds within which participants had to make a decision whether they would feel “extremely unpleasant” or “mildly unpleasant” from their own perspective.

After the scanning session, participants rated the unpleasantness of each action (7-step scale visual analogue Likert scale) in order to control for the degree of negative valence and emotional intensity. Furthermore, participants were required to “choose the feeling that (they) would feel most strongly” from a list of: guilt, contempt/disgust towards self, shame, indignation/anger towards self, indignation/anger towards other, contempt/disgust towards other, none, other feeling. As in our previous studies [19], [43], guilt and shame trials for the fMRI analysis were defined by individual ratings and they were restricted to agency-role-congruent responses (i.e. guilt and shame in the self-agency condition). This was because agency-role-incongruent responses occurred relatively rarely and may not be directly comparable with agency-role-congruent feelings. For example, feeling guilty for something one’s best friend has done would be mostly maladaptive and we wanted to restrict our analyses to adaptive “healthy” experiences of guilt in order to allow for a direct comparison of control and MDD group without confounding differences in the subjective experience. Likewise, only shame responses in the self-agency condition were modeled.

### Image Acquisition

Echo-planar T2*-weighted images (405 volumes in each of the 3 runs with 5 dummy scans for each run of 13 min 40 sec) were acquired on a Philips 3 Tesla Achieva MRI scanner with an 8 channel coil, 3 mm slice thickness and ascending continuous acquisition parallel to the anterior to posterior commissural line (between 35 and 40 slices depending on size of the participant’s head, Repetition Time (TR) = 2000 ms, Echo Time (TE) = 20.5 ms, Field of View (FOV) = 220×220×120 mm, acquisition matrix = 80×80, reconstructed voxel size = 2.29×2.29×3 mm, SENSE factor = 2). In addition 3-dimensional T1-weighted Magnetization-Prepared Rapid Acquisition Gradient Echo structural images were obtained (reconstructed voxel size = 1 mm^3^, 128 slices, TE = 3.9 ms, FOV = 256×256×128, acquisition matrix = 256×164, slice thickness = 1 mm, TR = 9.4 ms). Axial T2-weighted structural images were acquired for each participant to rule out vascular and inflammatory abnormalities.

### Analysis

Behavioural and supporting data analyses were performed using a significance threshold of p = .05, 2-sided (SPSS16, www.spss.com). Functional images were realigned, unwarped and coregistered to the subject’s T1 images. These images were normalized by first normalizing the participant’s T1 image to the standard T1-template in SPM8 (http://www.fil.ion.ucl.ac.uk/spm/) and applying the same transformations to the functional images. A smoothing kernel of FWHM = 6 mm was used. At the first (individual) level we contrasted shame vs. guilt and each of the moral emotions vs. fixation. In an exploratory model we also examined the self-agency condition including shame and guilt regressors convolved with self-agency and the other-agency condition including indignation/anger towards others regressors convolved with the other-agency condition. At the second level, we used shame vs. guilt and self-agency vs. other-agency contrast images in two different models. Using a two-sample t-test in our first model we compared the groups. Using a one-sample t-test in our second model, we aimed at detecting differences between conditions that were consistent across groups, by modeling group as a covariate of no interest. In secondary data analyses based on the means of activated clusters in the whole brain models (using MarsBar version 0.43, http://marsbar.sourceforge.net/ [44]), we confirmed that the detected regions did survive when comparing moral emotions vs. the low-level fixation baseline allowing us to infer increased activation for the moral emotion of interest rather than deactivation in the subtracted control emotion. We also ensured that observed effects were not driven by the subgroup taking medication. We further separated the groups and carried out supporting one-sample t-tests in order to examine whether group differences arose from activations for shame vs. guilt in one group or guilt vs. shame in the other.

Whole brain results were first explored at a voxel-level threshold of p = .005 uncorrected, 4 voxels. Only areas are reported that survived additional voxel- or cluster-level Family-Wise-Error (FWE)-corrected thresholds of p = .05 across a priori ROIs (as detailed below, small volume correction) or the whole brain. Supporting data analyses in each group used an FWE-corrected threshold of p = .10. A grey matter mask based on brains of all participants was used as an inclusive mask in all analyses [31].

### Region of Interest (ROI) Definition

Bilateral a priori ROIs used are further described in [31], [43]. We restricted the analysis to regions which were previously shown to be specifically related to guilt (ventromedial PFC including the septal/subgenual cingulate region and frontopolar cortex (BA 10, see eMethods section at http://archpsyc.jamanetwork.com/article.aspx?articleid=1171078, for further details on ROI construction) or which we hypothesized to be specific for shame (posterior superior temporal sulcus/temporo-parietal junction ROI and amygdala ROI). In order to show the specificity of our findings we also included control regions involved in moral emotions more generally [33], [34]: dorsolateral PFC, insula, basal ganglia, hypothalamus, ventral tegmental area, anterior temporal lobes and the medial temporal lobes highlighted in cortico-limbic network models of MDD [45].

## Results

### Behavioural Results

There were no differences between groups in the percentages of trials rated as guilt- or shame -evoking and no between-group differences on unpleasantness ratings or response times for guilt or shame trials (see Table 3). There were also no differences between groups on the percentage of “very unpleasant” response button choices during the fMRI scan (Control: 51.2±17.5%; MDD: 50.7±24.5%; t[37] = .07, p = .95).

**Table 3:** Summaries of moral emotion ratings and response times.

	Control	Remitted MDD	t-values	p-values
	mean ±SD	mean ±SD		
*Frequency (%)*				
guilt (self-agency)	29.1±10.5	27.5±8.9	. 50	.61
shame (self-agency)	21±10.8	15.7±10.2	1.5	.13
*Rated unpleasantness*				
guilt trials	4.5±.8	4.3±.6	1.15	.25
shame trials	4.4±1.1	4.5±.7	−.35	.72
*Response times (ms)*				
guilt trials	2317±572	2223±426	.57	.57
shame trials	2294±558	2111±414	1.15	.26

Summaries of between-group differences at p = .05, two-sided (MDD group: N = 21, control group: N = 18). Unpleasantness ratings were obtained on a 7-step visual analogue Likert scales (range 1 to 7). Ratings for guilt trials were reported previously [31].

Social behaviours in the negative self-agency condition that were described by negative concepts (e.g. “stingy”) were rated as more unpleasant and were more frequently associated with shame compared with those described by negated positive concepts (e.g. “not generous”) across both groups with no effect of group. This was tested using a repeated measures general linear model revealing a main effect of negation of concept on unpleasantness (F[1], [37] = 188.7, p<.0001) and shame (F[1], [37] = 11.5, p = .002) with no group by negation of concept interaction for unpleasantness (F[1], [37] = .5, p = .50) or shame (F[1], [37] = .2, p = .70). In contrast there were no differences in associated guilt for negated positive and negative concepts (main effect of negation of concept: F[1], [37] = 2.0, p = .16; group by negation of concept interaction: F[1], [37]<.0, p = .97).

### fMRI Results

**Figure 1:**
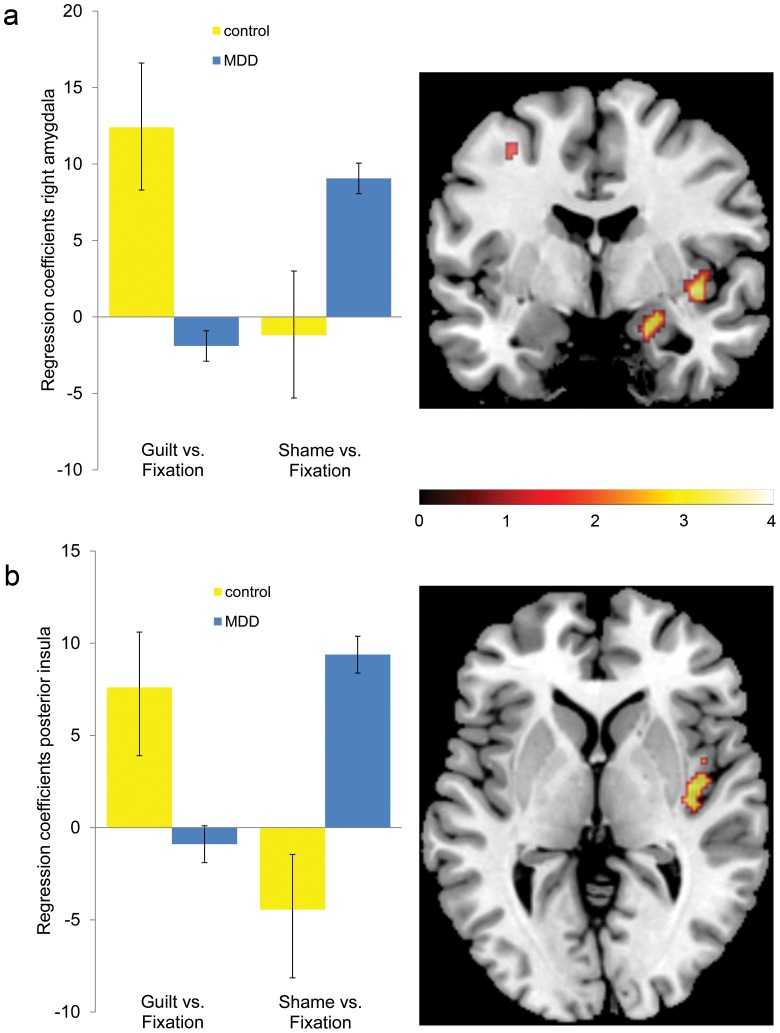
The MDD group showed higher activation in the right amygdala (panel a) and right posterior insula (panel b) for shame versus guilt compared with the control group (displayed are whole brain maps at voxel-level p = .005 uncorrected and cluster size of 4 voxels). This was confirmed by a supporting data analysis using the mean regression coefficients of the activated clusters in the amygdala (24, −4, −18) and posterior insula (40, −16, 0). For both regions, there was a moral emotion by group interaction (amygdala: F[1], [37] = 10.5, p = .003; posterior insula: F[1], [37] = 16.9, p<.0001) and no main effect of moral emotion (amygdala: F[1], [37] = .126, p  = .725; posterior insula: F[1], [37] = .11, p = .75) or group (amygdala: F [1], [37] = .30, p = .59; posterior insula: F [1], [37] = .79, p = .38). The increased shame-response relative to guilt compared with the control group was also found in the remitted MDD subgroup not currently taking medication (amygdala: p = .01, t[31] = 2.6; posterior insula: p<.0001, t[31] = 4.1).

When comparing shame vs. guilt, the MDD group showed greater activation within the right amygdala and right posterior insula than the control group (Table 4). Further analyses based on extracted regression coefficients from activated clusters showed that this effect was due to a group by moral emotion interaction in both regions such that the shame response in the MDD group was enhanced in both regions relative to the control group (see legend of Figure 1). Our supporting analyses separately for each group demonstrated that both regions showed enhanced activation for shame vs. guilt in the MDD group, but neither for guilt vs. shame, nor for shame vs. guilt in the control group. These effects were reproducible in the MDD subgroup not currently taking medication (see legend of Figure 1). There were no neural differences between shame and guilt conditions that were consistent across groups (Table 4).

**Table 4:** BOLD fMRI results.

Comparison	Contrast	Hemisphere	Region	X	MNIY	Z	t-value	FWE-corr. p-value
MDD>control	shame vs. guilt	R	amygdala	24	−4	−18	3.7	.05 ^c,1,^ *
		R	posterior insula	40	−16	0	4.0	.02 ^c,2,^ *
control>MDD	shame vs. guilt	–	no significant regions					
control & MDD	shame vs. guilt	–	no significant regions					
control & MDD	guilt vs. shame	–	no significant regions					

Only regions are reported that survived voxel- or cluster-based FWE-corrected p = .05 over the whole brain or our *a priori* ROIs. c = cluster-based FWE-correction. 1 = ROI with strong a priori predictions. 2 = Control ROI with weak a priori predictions. Control group: N = 18, remitted MDD group: N = 21.

* Additional analyses for each group separately showed that the amygdala and posterior insula were activated for shame vs. guilt in the MDD group, but not for guilt vs. shame in the control group (at FWE-corrected p = .10 over ROIs).

We also explored the contrasts self-agency vs. other-agency and other-agency vs. self-agency in all participants, as well as between groups. There was only one comparison resulting in significant effects that were driven by activation increases in the condition of interest rather than decreases from the fixation baseline as determined by extracted regression coefficients: The left temporo-parietal junction (x = −42, y = −58, z = 20) showed higher activation for self-agency vs. other-agency in the control group compared with MDD (cluster-based FWE-corrected p = .03 over temporo-parietal junction ROI).

We further examined the relationship of posterior insula and amygdala activation with the unpleasantness of shame experiences in each individual. Individual averages of rated unpleasantness for shame trials were not associated with amygdala responses for shame vs. guilt (Control: Pearson’s r = .39, p = .11, N = 18; MDD: r = −.03, p = .91, N = 21, after excluding outliers outside of 2.5 SDs from each group’s mean: Control: r = .18, p = .49, N = 17; MDD: r = .−03, p = .90, N = 20). However, there was a significantly positive correlation of unpleasantness of shame trials with posterior insula signal for shame vs. guilt in the control (r = .55, p = .02, N = 18; after exclusion of outliers: r = .45, p = .07, N = 17), but not the MDD group (r = .24, p = .29, N = 21). A general linear model using outlier-excluded posterior insula signal for shame vs. guilt as the outcome variable (F[4], [37] = 5.5, p = .002, R-square = .41), showed that there was no significant group by unpleasantness of shame trials interaction (F[1], [37] = 2.2, p = .15), and that there were no main effects of unpleasantness of shame trials (F[1], [37] = 2.1, p = .16) or of guilt trials (F[4], [37] = .8, p = .40), whilst confirming the expected main effect of group (F[4], [37] = 4.6, p = .04). These results supported the conclusion that between-group differences in neural activity in the amygdala and posterior insula cannot be explained by differences in the unpleasantness of shame or guilt experiences.

## Discussion

We confirmed our general hypothesis that people with MDD exhibited enhanced shame-selective activation in brain regions linked to the sensory perception of emotions. This was based on evidence that shame, unlike guilt, requires an imagined critical observer [7], [8] and on previously shown activations of sensory areas when engaging in mental imagery [28], [29]. Our more specific predictions were only partly confirmed in that indeed the amygdala showed shame-selective activation in the MDD group, but that there was no difference within the posterior superior temporal or temporo-parietal region between shame and guilt. Instead, we found an unexpected shame-selective activation increase in the right posterior insula in the MDD compared with the control group. Whilst this was unexpected, these findings are in general agreement with our hypothesis that shame is associated with higher activations in regions linked to sensory perception of emotionally relevant stimuli.

Our finding of a shame-selective increase in amygdala-response concurs with the hypothesis of a distinctive role of shame in MDD relative to guilt. The amygdala plays a prominent role in neural models of MDD [45]–[48]. Metaanalytic reviews confirm that the amygdala is more responsive to sensory stimuli than to internally generated emotional responses [38]. Amygdala activations were reliably associated with sensory perception of emotionally and socially relevant materials [35]–[37]. The amygdala was also shown to be activated when simulating the pain experiences of another person based on images of facial expressions of pain [49]. Shame-selective amygdala responses are therefore in keeping with the notion that mental imagery requires simulated sensory perception [29] and that shame depends more strongly on simulated perception by others than guilt [27].

Increased amygdala activations were reproducibly found in people with current MDD when presented with negative emotional material [50], [51]. Some studies found increased amygdala responses to sad faces to be present in remitted MDD as well [52], [53]. A growing body of evidence, however, suggests normalization in amygdala response to facial expressions of emotions on remission [54]–[57], which in one study may have been related to the effects of antidepressant medication rather than remission itself [53]. However, overall normal levels of amygdala activation could result from between-subject differences. One study showed, for example, that although amygdala activation was not elevated consistently in a group of remitted MDD, its activation predicted subsequent recall of negative self-referential memories [58]. By using random-effects models in our analyses, we demonstrated that shame-selective increases in amygdala activation were consistent across individuals with MDD compared with the control group. Our finding of increased amygdala-response to self-related negative emotions is also in keeping with the view that MDD is associated with neural changes related to increased negative self-focus [59] involving the amygdala [60].

Our finding that guilt did not activate the amygdala to significant degrees in either group is in keeping with the evidence derived from healthy control samples and MDD reviewed in the introduction. However, a recent fMRI study in healthy volunteers reported the amygdala to be activated for guilt compared with shame [61]. A closer inspection of this finding reveals, however, that the peak of the large cluster of activation entailing the amygdala was located within the thalamus.

The posterior insula was not reported in metaanalyses of fMRI activation studies in MDD [50], [51] [62], [63]. However, there is evidence that posterior insular hypermetabolism predicts treatment response [64]. Several lines of evidence suggest the posterior insula carries primary representations of emotionally relevant somato-sensory signals [65]. It is specifically connected to sensory-motor cortices [66] and is implicated in primary pain [67], temperature and touch perception – particularly the perception of affiliative touch [68], [69]. Affiliative touch is one of the ontogenetically earliest ways of bonding with others [70] and shame entails the anticipated rejection of others [71]. That shame scenarios also engage posterior insula in MDD is thus compatible with the hypothesis that shame-proneness is related to sensory experiences when simulating an external observer [27].

Except for a non-predicted activation increase in the temporo-parietal junction for self-agency vs. other-agency in the control group compared with MDD, there were no significant group differences for this contrast. This is in concordance with our previously reported BOLD results for guilt vs. indignation/anger towards others [31].

On a more cautionary note, shame-selective increases in amygdala and posterior insula response could be linked to different types of vulnerability traits for MDD. One possibility is that they are due to scarring effects of previous episodes [72]. Another possibility is that they are associated with primary vulnerability before the onset of MDD. Studies in high-risk groups, such as people with a family history but without a previous personal history of MDD may help in distinguishing primary from secondary vulnerability. Furthermore, our finding regarding the posterior insula needs confirmation because it was based on a control ROI with weak a priori rather than one of our ROIs with strong a priori predictions.

Because of the variability in shame-proneness, there were some participants with a low number of trials available for analysis potentially limiting the statistical power to detect effects. The reported group differences, however, cannot be explained on this basis, as the groups did not differ on the number of shame trials. One could argue that differences in how the groups remembered their emotional response during the scan could have affected their post-scan ratings. It is unlikely, however, that a bias in remembering emotional responses would affect guilt and shame in systematically different ways.

Further, some clinical characteristics of our MDD group need further consideration. Although, the majority of patients in our MDD group had only experienced one previous episode, they nevertheless had a largely increased life-time risk of developing another episode compared with the control group (approximately 50% vs. 15% [73]). This study was deliberately designed to exclude patients with MDD and relevant other axis-I disorders. Therefore our results may not be generalizable to patients with MDD and co-morbid other axis-I disorders.

We opened this paper with a quote by Jean-Jacques Rousseau. From a philosophical perspective, the quote illustrates the extent to which guilt and shame are intertwined emotions. From a medical perspective, Rousseau argues that under certain circumstances guilt may share some of the physiological reactions (i.e. blushing) which are purported to accompany feelings of shame. Despite the large overlap between guilt and shame as suggested by Rousseau, our study found that limbic brain regions distinguish between them.

### Conclusion

We demonstrated that people with MDD exhibited an increased response to shame within the right amygdala and posterior insula, when compared with the control group. This increased shame response was selective relative to guilt. The results were not due to differences in perceived emotional intensity between the groups. Further, group differences were not due to effects of antidepressant medication. This supports the hypothesis that shame and guilt play distinct roles in vulnerability to MDD. Future studies are needed to directly compare shame-induction with facial emotion recognition paradigms. Our results indicate that shame-induction may be a more powerful probe of residual amygdala hypersensitivity in MDD after symptoms have subsided. This has important implications for designing imaging biomarkers of recurrence risk in MDD.
